# Comparative Proteomic Analysis of Walnut (*Juglans regia* L.) Pellicle Tissues Reveals the Regulation of Nut Quality Attributes

**DOI:** 10.3390/life10120314

**Published:** 2020-11-27

**Authors:** Paulo A. Zaini, Noah G. Feinberg, Filipa S. Grilo, Houston J. Saxe, Michelle R. Salemi, Brett S. Phinney, Carlos H. Crisosto, Abhaya M. Dandekar

**Affiliations:** 1 Department of Plant Sciences, University of California, Davis, CA 95616, USA; pazaini@ucdavis.edu (P.A.Z.); ngfeinberg@ucdavis.edu (N.G.F.); hsaxe@ucdavis.edu (H.J.S.); chcrisosto@ucdavis.edu (C.H.C.); 2 Department of Food Sciences and Technology, University of California, Davis, CA 95616, USA; fsgrilo@ucdavis.edu; 3 Proteomics Core Facility, University of California, Davis, CA 95616, USA; msalemi@ucdavis.edu (M.R.S.); bsphinney@ucdavis.edu (B.S.P.)

**Keywords:** walnut kernel, pellicle color, LC-MS/MS proteomics, kernel quality, plant genome-wide proteome, small heat shock proteins, fatty acid metabolism, seed coat pigmentation

## Abstract

Walnuts (*Juglans regia* L.) are a valuable dietary source of polyphenols and lipids, with increasing worldwide consumption. California is a major producer, with ’Chandler’ and ’Tulare’ among the cultivars more widely grown. ’Chandler’ produces kernels with extra light color at a higher frequency than other cultivars, gaining preference by growers and consumers. Here we performed a deep comparative proteome analysis of kernel pellicle tissue from these two valued genotypes at three harvest maturities, detecting a total of 4937 *J. regia* proteins. Late and early maturity stages were compared for each cultivar, revealing many developmental responses common or specific for each cultivar. Top protein biomarkers for each developmental stage were also selected based on larger fold-change differences and lower variance among replicates, including proteins for biosynthesis of lipids and phenols, defense-related proteins and desiccation stress-related proteins. Comparison between the genotypes also revealed the common and specific protein repertoires, totaling 321 pellicle proteins with differential abundance at harvest stage. The proteomics data provides clues on antioxidant, secondary, and hormonal metabolism that could be involved in the loss of quality in the pellicles during processing for commercialization.

## 1. Introduction

Walnuts, the seed of trees from the genus *Juglans*, are prized among the edible nuts for their unique quality attributes, visual appearance, taste, texture, and nutritional components. Walnuts have grown in popularity around the globe due in large part to their perceived health benefits [1,2,3,4] stemming from their high concentrations of polyphenols, vitamin E (α-tocopherols) antioxidants, and essential polyunsaturated fatty acids [5,6,7]. While not fully understood, current research indicates that some of these bioactive compounds present in walnuts could provide cardiovascular and cognitive health-protective benefits [3,4,8,9,10,11]. Demand for walnuts is highest in China, comprising nearly half of all global walnut consumption, followed by Iran, the United States, and Turkey [12]. In terms of production, China is the global leader followed by the United States with the California walnut industry accounting for nearly all domestic production, generating an annual revenue of $1.28 billion and producing 653,000 tons as of 2019 [13,14,15].

Walnut kernels are commercially graded based on kernel pellicle color, nut size, and shell thickness. In addition to orchard management factors, cultivars vary in their ability to produce extra-light kernels. The primary walnut cultivar grown in California is ‘Chandler’, which shows a high frequency of light-toned kernels and comprises approximately 40% of the total production, followed by ‘Hartley’, ‘Howard’ and ‘Tulare’, each with about 10% of the total production. ‘Howard’ and ‘Tulare’ were produced and selected by the University of California, Davis walnut breeding program because they are mid-late season harvest with large nut sizes and suitable for in-shell marketing. These two cultivars’ volume is increasing, especially in the Northern California production area as they are harvested before ‘Chandler’, thus, extending the harvesting season.

While commercially classified as nuts, walnuts are botanically classified along with almonds (*Prunus dulcis*) and pistachios (*Pistacia vera*) as drupaceous nuts, a kind of indehiscent fruit that does not open to expose the seed when mature. The edible portion of the walnut is the kernel, comprised of both the meat (embryo and endosperm) and the pellicle/seed coat (ovular integument), which are further enclosed within a layer of packing tissue and a hard shell (endocarp), and finally the green fleshy hull (mesocarp and exocarp) [16,17,18,19]. Walnuts reach commercial maturity and highest quality at “packing tissue brown” (PTB), where the packing tissue surrounding the kernel dries and the vascular connections to the tree cease to function [20]. Following the onset of PTB, the kernel continues to age as the outer hull begins to rapidly mature through the harvest maturity stages M1 (“Intact Hull”), M2 (“Hull Split”), and M3 (“Hull Bloom”), with commercial harvest occurring between M2 and M3. The terms “Hull Split” and “Hull Bloom” refer to the progressive stages of hull dehiscence, by which the fleshy green hull splits, desiccates, and pulls away from the walnut shell. This process facilitates mechanical harvest, which begins in California between September and November, followed by hull removal (for nuts with incomplete hull dehiscence), drying, and grading for shelled walnuts. In California, walnut kernels are assessed for quality following the California Dried Fruit Association (DFA) standards based on kernel size and pellicle color, with “premium quality” being defined as large-sized and light-colored kernels with no “off flavor” [21]. Walnuts are visually evaluated based on kernel color and subjectively assigned grades of “Extra Light”, “Light”, “Amber”, and “Dark Amber”. Consumer preference leans heavily towards lighter-colored kernels, and industry growers are compensated for their crop based on quality, leading to significant economic losses on a season’s crop due to color problems [22]. This color problem originates and is restricted to the walnut pellicle (seed coat), and despite the importance of this phenotype, little is known about its underlying physiology [16].

The seed coat serves a wide range of critical roles for the developing and mature embryo and endosperm. These include physical protection of the seed from its environment and pathogens, a barrier to oxygen, perception of environmental cues, a placenta-like connection between developing seed and mother plant, and control of seed dormancy, among others [18,23]. The seed coat in walnut has been shown through transmission electron microscopy to function transporting nutrients from the mother plant to the developing embryo [23]. Additionally, the seed coat regulates critical quality attributes as it accumulates most of the antioxidant phenolic compounds present in the walnut kernel, supporting its role as a barrier to pests and oxygen that may induce damage to the embryo [24,25].

Molecular studies into the walnut pellicle physiology related to various quality attributes are limited and recently were incremented by methods employing genome-wide quantitative trait loci (QTL) and single nucleotide polymorphisms (SNP) [26,27,28]. A reference genome sequence established the conditions necessary for in-depth proteomic analyses as it provided individual protein sequences at a genomic scale, considerably improving the identification of detected proteins [29,30]. Knowledge on walnut kernel physiology has also benefitted from a metabolomics study showing that nitrogen metabolism is essential for maturation, during which carbon from carbohydrates and proteins is transferred into other compounds such as fatty acids during kernel ripening [31]. The bulk of the research on the walnut pellicle has been documentation of quality loss from darkening that reduces the visual appeal for consumers. Besides environmental factors that fluctuate annually, the most important and controllable factors for developing this quality attribute appear to be cultivar and harvest timing [20,32,33,34]. After the kernel reaches maturity at PTB, there is a time delay until the outer hull reaches the maturities (M2 and M3) that enable efficient harvest. Delayed harvest from M2 to M3 can decrease the percentage of high-quality kernels after commercial harvest and drying, making harvest timing a crucial factor to consider for maintaining kernel quality.

Despite its significant effect on quality, the molecular mechanisms within pellicle tissue during this period of delayed harvest are still not fully understood. Here we performed an in-depth proteomic analysis of isolated walnut pellicle tissues for elite cultivars ‘Chandler’ and ‘Tulare’ at the harvest maturity stages M1, M2, and M3. Our data shows that most of the detected proteome is shared between the cultivars and maturity stages, but also reveals specific protein biomarkers for each sample type. These findings contribute to our understanding of proteins involved in the early stages of pellicle color development during pre-harvest conditions that affect postharvest darkening.

## 2. Materials and Methods

### 2.1. Plant Material

Walnut samples were collected during the 2018 season from a commercial orchard of ‘Tulare’ and ‘Chandler’, planted in 2013 in Wheatland, CA (39°00′36.7″ N/121°28′06.5″ W). A plot of each cultivar with 500 trees was planted at a density of 400 trees/ha (5 m × 5 m), grown using full-coverage micro-sprinklers, and applied standard irrigation to keep the trees at no less than ~0.2 MPa below the proposed walnut baseline water status while avoiding prolonged periods at or wetter than the baseline. From each plot 30 trees were randomly selected and labeled for collecting physiological data and sampling.

### 2.2. Physiological Maturity Sampling

**Figure 1:**
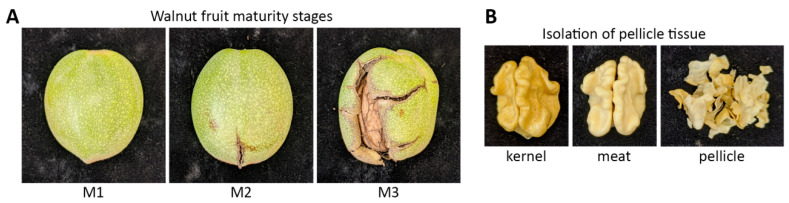
Representation of samples used in this study. (**A**) Walnut fruit maturity stages. Intact Hull (early, M1), hull split (intermediate, M2), and hull bloom (late, M3). These stages are determined by the level of the hull (exocarp) split; (**B**) Isolation of pellicle tissue used for proteomic analysis. The commercial product (kernel) comprises the meat (walnut embryo + endocarp) surrounded by the pellicle tissue that develops a darker color. Pellicle tissues were separated from the meat and used for proteomic analysis.

Samples from both cultivars were harvested at three physiological maturities based on the PTB date [22]. PTB was determined by randomly collecting 100 nuts from the top and the bottom of the trees every week until 100% of walnuts showed packing tissue turning brown [35]. When the inner side of the hull reached 100% PTB, walnuts were at maturity 1 (M1); maturity 2 was at the onset of hull split (M2), and at full hull dehiscence, walnuts were at maturity 3 (M3) (Figure 1A). ‘Chandler’ and ‘Tulare’ reached 100% PTB on 10 September and 12 September, respectively. M1 was harvested on 17 September, M2 was harvested on 18 September, and M3 was harvested on 19 September. At each maturity stage, 90 nuts were harvested from 30 trees. Twenty walnuts were subsampled for pellicle extraction and thirty walnuts for kernel color evaluation at harvest per each cultivar-maturity.

Each of the ten pellicle replications contains the embryo pellicles of two walnut kernels. Each replicate with about 1 g of fresh tissue was immediately flash-frozen in liquid nitrogen and then stored at −80 °C until protein extraction. The remaining in-shell nuts were dried at 43 °C until they achieved 8.0% moisture content, determined by weight. After drying, nuts were cracked carefully by hand, using small hammers and tweezers to extract undamaged halves (Figure 1B). Of the walnuts harvested for each replicate, nuts were cracked randomly until there were 10 sound halves, without insect damage, shriveling, or other damage unrelated to the treatment. The kernel halves were placed in plastic walnut evaluation trays obtained from the California Dried Fruit Association. These trays have 100 slots, sized to hold kernel halves. The trays were spray-painted black to provide a uniform background color for the color evaluation. The walnuts were stored in trays inside cardboard boxes at 0 °C until they were evaluated for initial kernel color evaluation, within one week of cracking.

### 2.3. Walnut Kernel Color Evaluations

Individual kernel color was evaluated using two different methods: a subjective scaled score following current Dried Fruit Association (DFA) guidelines (United States Standards for Grades of Shelled Walnuts 2017); and a Minolta Colorimeter (Konica Minolta, Ramsey, NJ, USA). The DFA provides a chart for color evaluation that places the kernels into one of four categories: extra light, light, light amber, and amber. The Minolta Colorimeter represents color as L (lightness) and hue° (color shade) [36]. The color of each individual kernel was measured positioning the Minolta Colorimeter in the center of the outer side of the walnut kernel half (closest to the shell) according to their placement in the DFA trays at harvest according to [37]. Pellicle color data was subjected to an analysis of variance (ANOVA), correlation analysis and the means were separated using Tukey’s test (*p* ≤ 0.01 or *p* ≤ 0.05) using the R statistical program [38]. Additionally, 51,100 ‘Chandler’ and 2900 ‘Tulare’ kernels from various orchards in Northern California were evaluated in 2019 by Scientific Methods, Inc. (Beckley St, Honolulu, HI, USA) using the DFA guidelines from the United States Food and Agriculture walnut color chart [21].

### 2.4. Walnut Pellicle Protein Extraction and Preparation of Samples for Mass Spectrometry

Protein extraction was performed based on a previously described phenol extraction protocol [39] with modifications. Ten replicates of each of the six sample types (three maturity stages of cultivars ’Chandler’ and ’Tulare’) were used for proteomic analysis. Pellicle tissue was peeled from kernels with tweezers, and frozen immediately with liquid nitrogen, and stored at −80 °C. Frozen pellicle tissue was ground to a fine powder with a mortar and pestle in liquid nitrogen. About 150 mg of fine frozen powder was suspended in 3 mL extraction buffer (500 mM Tris-HCl, 50 mM EDTA. 700 mM sucrose, 100 mM KCl, 2% β-mercaptoethanol, 1 mM PMSF, and SigmaFAST™ Protease Inhibitor Cocktail, Sigma-Aldrich, St. Louis, MO, USA). The suspension was homogenized and incubated on ice under shaking at 100 rpm for 10 min. An equal volume of Tris-buffered Phenol pH 7.0 was added and homogenized on a shaker at 100 rpm at room temperature for 10 min. The samples were centrifuged at 5500× *g* for 10 min at 4 °C and the upper phenol phase was carefully recovered and poured into a new tube. The phenol phase was re-extracted in 3 mL of extraction buffer, followed by homogenization by vortexing for 3 min, and centrifugation following the sequence used previously for phase separation. The new phenol phase was carefully recovered and poured into a new tube. Proteins were precipitated using four volumes of cold precipitation solution (100 mM ammonium acetate in methanol) overnight at −20 °C, followed by centrifugation at 5500× *g* for 10 min at 4 °C. Protein pellets were washed three times with 1 mL cold precipitation solution and one time with 300 µL cold acetone. The pellet was dried for 1 h in the hood and 1 min under vacuum. Proteins were solubilized with 150 µL urea buffer (6 M urea and 50 mM triethylammonium bicarbonate). For protein quantification, 10 µL was used for a 1:10 dilution to determine the protein concentration using the Pierce™ BCA Protein Assay Kit (Thermo Fisher Scientific, Waltham, MA, USA) with bovine serum albumin (BSA) as a standard. Protein digestion and tandem mass tagging (TMT) labeling (Thermo Fisher Scientific, Waltham, MA, USA) was carried out with 100 µg crude protein extract from each sample according to a previously described procedure [40].

**Figure 2:**
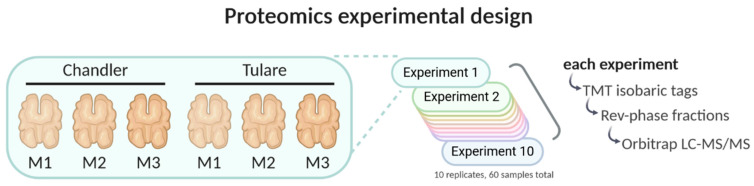
Proteomics experimental design. Kernel pellicle samples from cultivars ‘Chandler’ and ‘Tulare’ from three maturity stages (M1, M2, and M3) were used. Ten replicates of each sample type were used in total. Each mass spectrometry experiment consisted of one sample from each of the six sample types labeled with isobaric tags for quantitative comparison. Each experiment was reverse-phase fractionated in 8 samples according to hydrophobicity for deeper identification using Orbitrap Lumos LC-MS/MS. Created with Biorender.com.

Liquid chromatography followed by tandem mass spectrometry (LC-MS/MS) was performed as follows. LC separation was done on a Dionex nano Ultimate 3000 (Fisher Scientific, Waltham, MA, USA) with a Thermo Easy-Spray source. The high pH separated peptides were reconstituted in 2% acetonitrile/0.1% trifluoroacetic acid and 5 µL of each sample was loaded onto a PepMap 100 Å 3U 75 µm × 20 mm reverse phase trap where they were desalted online before being separated on a 100 Å 2U 50-micron × 150 mm PepMap EasySpray reverse phase column. Peptides were eluted using a 120-min gradient of 0.1% formic acid (A) and 80% acetonitrile (B) with a flow rate of 200 nL/min. The separation gradient was ran with 5% to 8% B over 3 min, 8% to 10% B over 3 min, 10% to 24% B over 80 min, 24% to 50% B over 17 min, 50% B to 99% B over 5 min, a 2-min hold at 99% B, and finally 99% B to 2% B held at 2% B for 5 min. The MS3 Synchronous Precursor Selection Workflow was as follows. Mass spectra were collected on a Fusion Lumos mass spectrometer (Thermo Fisher Scientific) in a data dependent MS3 synchronous precursor selection (SPS) method. MS1 spectra were acquired in the Orbitrap, 120 K resolution, 50 ms max inject time, 5 × 105 max inject time. MS2 spectra were acquired in the linear ion trap with a 0.7 Da isolation window, collision-induced dissociation fragmentation energy of 35%, turbo scan speed, 50 ms max inject time, 1 × 104 automatic gain control (AGC) and maximum parallelizable time turned on. MS2 ions were isolated in the ion trap and fragmented with a higher energy collisional dissociation of 65%. MS3 spectra were acquired in the orbitrap with a resolution of 50 K and a scan range of 100–500 Da, 105 ms max inject time and 1 × 105 AGC. Figure 2 illustrates the sample types used in each of the ten experiments and a summary of their preparation for proteomic analysis.

### 2.5. Proteome Data Preparation and Analysis

The Thermo binary instrument files were processed with MSConvert from the Proteowizard toolkit [41] and Python scripts from the PAW (Proteomic Analysis Workflow) pipeline https://github.com/pwilmart/PAW_pipeline [42] to create MS2-format files [43] for database searching and to extract the reported ion peak heights from individual MS3 scans. Database searching used Comet version 2016.01 rev. 3 [44] with the PAW pipeline for peptide spectrum match (PSM) validation using the target/decoy method [45]. Search parameters included: parent ion monoisotopic mass tolerance 1.25 Da, fragment ion monoisotopic mass tolerance 1.0005 Da, trypsin cleavage with up to two missed cleavages, variable oxidation of Met residues, fixed alkylation of Cys residues, and fixed TMT reagent masses (+229.1620 Da) at peptide N-terminus and Lys residues. The protein database of *Juglans regia* (41,103 sequences downloaded from NCBI assembly GCF_001411555.2) was used to map the identified peptides. Common contaminants (179 sequences) and sequence-reversed decoys were also appended. Peptide-spectrum matches were filtered to a 1% false discovery rate (FDR) and mapped to proteins using basic parsimony rules. Protein identifications required at least two distinct PSMs per protein per TMT plex. An additional extended parsimony protein grouping step was used to combine highly homologous proteins into the final list of identified proteins. Shared or unique peptide status was defined in the context of the final protein list. Reporter ion intensities from PSMs associated with unique peptides were summed into protein total intensity values. Protein intensities within each TMT plex and between plexes were normalized using the Internal Reference Scaling (IRS) method https://github.com/pwilmart/IRS_normalization [46] on a virtual reference pool generated from averaging the TMT intensity signal for each protein in each TMT set. Proteins with differential abundance were determined by calculating Benjamini-Hochberg adjusted *p*-values (FDR correction for multiple comparisons) on *p*-values obtained from T-test using ten replicate samples of the two conditions being compared. Raw data and complete PAWS pipeline results is are available at both the Massive proteomics repository https://massive.ucsd.edu/ [47] and Proteome exchange http://proteomexchange.org/ [48] under repository numbers MSV000086493 and PXD022655, respectively. Gene ontology (GO) analysis was performed in the PANTHER server http://www.pantherdb.org/ [49] using GO annotations of *Vitis vinifera* as a reference. Multivariate analysis was performed with metaboanalyst.ca 4.0 [50] without further normalization or filtering, and auto data scaling.

## 3. Results

### 3.1. Analysis of Walnut Pellicle Coloration

**Figure 3:**
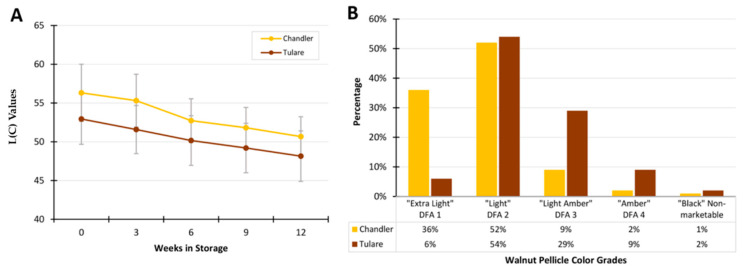
Colorimetric analysis of ‘Chandler’ and ‘Tulare’ kernels. (**A**) Average and standard deviation of Minolta L(C) values for kernels stored at 25 °C for 12 weeks (*n* = 150 kernels per cultivar were evaluated); (**B**) Proportion of postharvest kernel darkening incidence for ‘Chandler’ and ‘Tulare’ cultivars of English walnut. All kernels used for pellicle extraction used in the proteomics analysis were from color grades DFA1 and 2 (*n* > 2000 kernels per cultivar were evaluated, see Methods). Dried Fruit Association (DFA) grades according to USDA walnut color chart.

In this work, we studied the maturity stages M1, M2, and M3. These maturities represent the initial stages of pellicle development following PTB and refer to hull tissue integrity. Although all the kernels used in this work were of similar color visually, belonging to the “extra light” or “light” color grades, small non-significant differences were detected by the Minolta imaging equipment. The color L(C) values reduced slightly throughout kernel storage following harvest, as shown in Figure 3A. Although kernels of similar color grades were used for the proteomics analysis presented next, it is important to note that these two cultivars produce kernels at different frequencies of the DFA color grades. Figure 3B shows the superior performance of the elite cultivar ‘Chandler’ when a larger number of nuts are evaluated. At harvest, ‘Chandler’ produces a much larger proportion of kernels in the “extra light” category than ‘Tulare’, which produces kernels predominantly in the “light” category. These proportions are relevant if samples representing the cultivar at the point of sale are to be analyzed.

### 3.2. Proteomic Analysis of Walnut Pellicle Tissues

**Figure 4:**
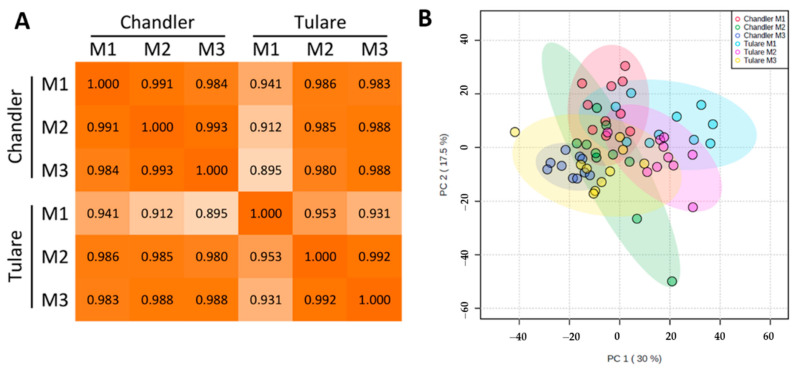
Multivariate analysis of proteome data. (**A**) Pearson correlations among the six sample types used for proteomics; (**B**) Principal Component Analysis showing clustering of samples used for proteomic analysis colored by sample type: ’Chandler’ M1 (red), M2 (green), M3 (blue), and ’Tulare’ M1 (cyan), M2 (pink), M3 (yellow). Ellipses denote 95% confidence intervals.

To assess the effect of walnut fruit maturation and genotype on pellicle color, we performed a quantitative proteomic analysis on pellicle samples collected at 3 different maturity stages (M1, M2, and M3) from two cultivars. ‘Chandler’ retains mostly an extra light color (higher market value) and ‘Tulare’ displays a lower incidence of extra light kernels compared to ‘Chandler’. A total of 4937 proteins were identified in at least one sample, and of these, 2800 were identified in all 60 samples (ten replicates of each sample type) (Table S1). Although overall correlation is high among all sample types, ‘Chandler’ samples retain a higher correlation among maturity stages than ‘Tulare’, based on individual protein levels (Figure 4A). Principal component analysis (PCA) shows that maturity stage (M1, M2, and M3) exerts a substantial effect on the clustering of samples from both genotypes (Figure 4B), and the samples have enough similarity to prevent formation of distinct groups.

### 3.3. Effect of Walnut Fruit Maturity on Kernel Pellicle Proteome

Besides the obvious phenotypic changes observed in the fruit exocarp (hull) tissues among the sampled maturation stages shown in Figure 1, other less apparent changes also occur in internal tissues, such as kernel pellicle darkening. To better understand the metabolic changes that precede and facilitate this process, walnut pellicle tissue from two of the major commercial varieties, ‘Chandler’ and ‘Tulare’, were analyzed. Initial valuable information that can be extracted from this analysis is the subset of proteins displaying the highest abundance in pellicle tissues. These include five 11S globulin seed storage proteins (Jr10_24240, Jr01_32050, Jr01_32060, Jr07_23180, and Jr01_32040), two vicilin-like antimicrobial proteins (Jr07_24930 and Jr05_09660), non-specific lipid-transfer protein-1 (Jr03_26990), sucrose-binding protein (Jr08_13930), and mannitol dehydrogenase (Jr08_18850). The complete set is shown in Table S1. When sorted by total protein abundance, these proteins’ massive contribution to the pellicle proteome becomes evident.

To maximize the differences accumulated during the nut maturation process, protein levels in the late maturity stage (M3) were compared to those observed in the early maturity stage (M1). Considering our quality control filters of a minimum of two peptides mapped to any given protein for it to be considered as “detected”, proteins with significant differential abundance (FDR < 0.05 among the 10 replicates of late vs. early maturity nuts) were determined (Table 1).

**Figure 5:**
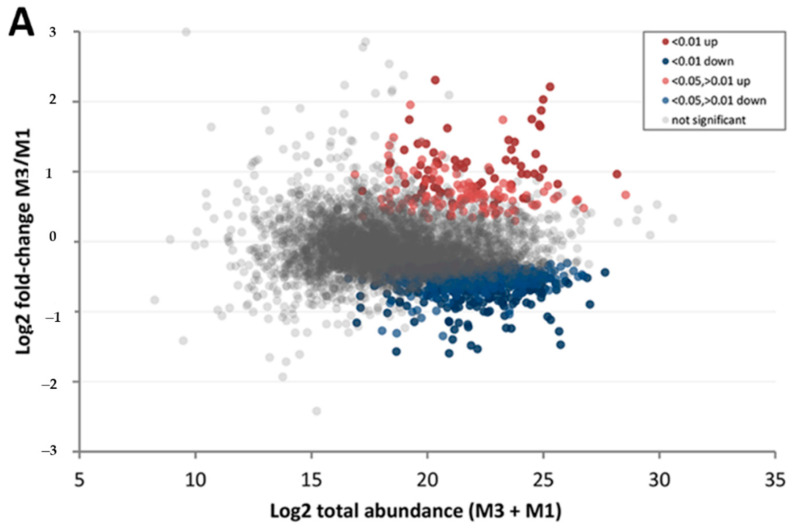

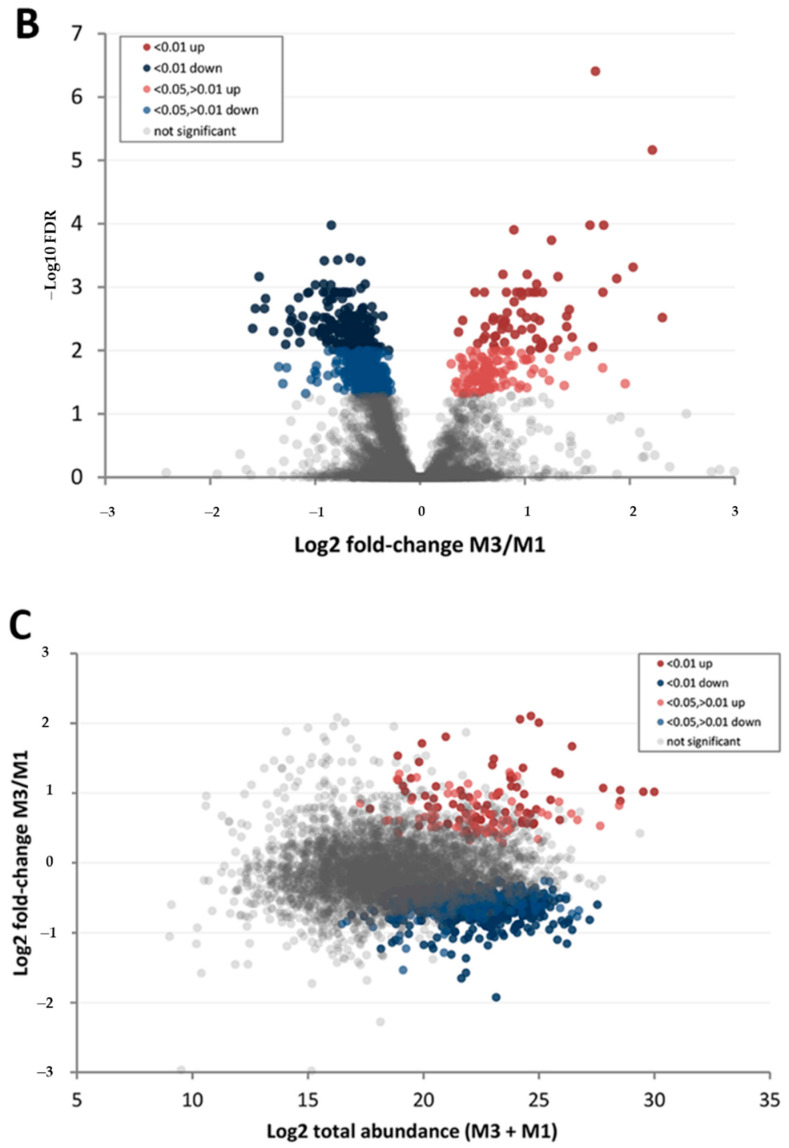

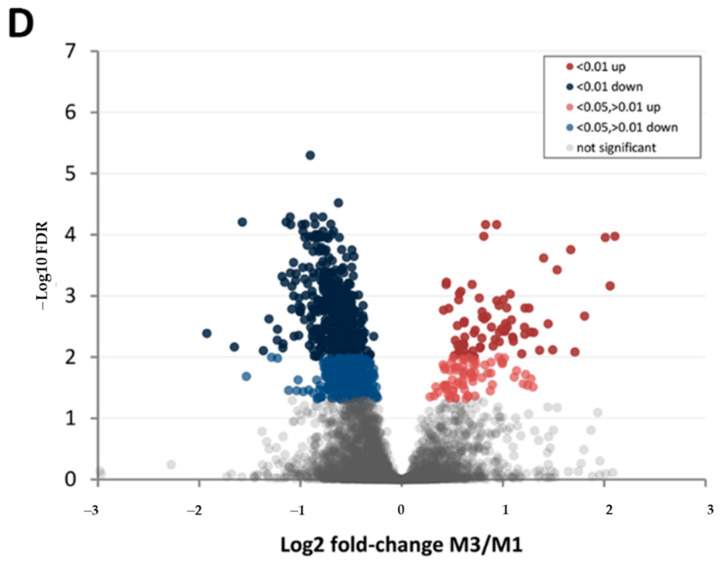
Representation of ‘Chandler’ and ‘Tulare’ proteomes during fruit maturation. (**A**) Ratio vs. abundance plot of cultivar ‘Chandler’ comparing late (M3) and early (M1) maturity stages, as listed in Table 1. Each dot represents the abundance of a protein, colored by the significance of the difference between M3 and M1. High confidence values (FDR < 0.01) are colored dark red (higher in M3) or dark blue (higher in M1), and medium confidence value (FDR < 0.05, >0.01) in lighter red and blue tones. Proteins without significant difference between M3 and M1 stages are colored gray; (**B**) Volcano plot showing M3 and M1 fold-change ratios of ‘Chandler’ proteome sorted by confidence levels, using the same color scheme; (**C**) Similar ratio vs. abundance plot of cultivar ‘Tulare’; (**D**) Volcano plot for ‘Tulare’ M3 vs. M1 samples.

While most of detected proteins did not show a significant difference between late and early maturity stages, a higher proportion of those with significant differences show reduced abundance in the late maturity stage on both cultivars, possibly due to a lower metabolic activity as maturation progresses. This trend is observed both on the group of proteins with differential abundance considered “high confidence” (FDR < 0.01) and those considered “medium confidence” (FDR between 0.05 and 0.01). Proteins detected with differential abundance but with FDR higher than 0.05 were not considered as differentially abundant in the comparisons. Complete sets of this differential abundance analysis are shown in Table S2. A stringent threshold of FDR < 0.01 for “high confidence” was used given the high number of replicates (ten) for each sample type. Protein abundance spanned six orders of magnitude, and its effect on abundance ratio and confidence level is shown in Figure 5A,B for cultivar ‘Chandler’ and Figure 5C,D for ‘Tulare’. No specific threshold was applied on the fold-change ratio to avoid false-negative results, but the confidence filter (FDR < 0.05) does affect the minimum fold-changes considered significant, as can be seen on Figure 5, both for ‘Chandler’ and ‘Tulare’ cultivars.

**Figure 6:**
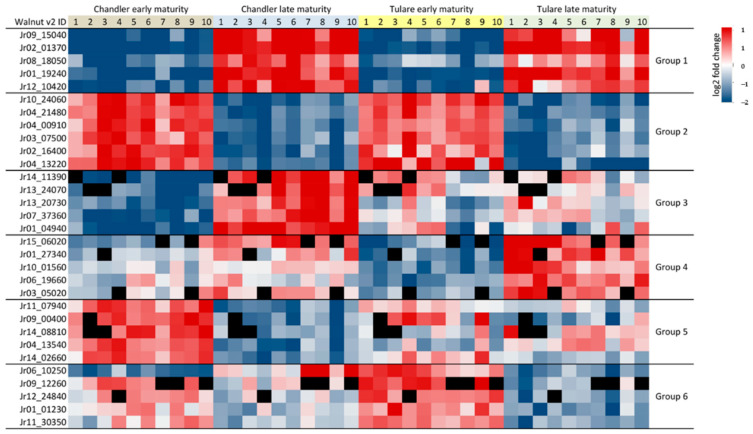
Heatmap of protein abundance ratios comparing early and late maturity stages, for cultivars ‘Chandler’ and ‘Tulare’. The ten replicates for each of the four sample types are shown, and individual ratios were calculated using the average of the condition compared. Missing values for a particular replicate are shown in black. Protein markers in the lower abundance range are sometimes not detected in a particular replicate but given the 10 replicates used still have enough data for the difference between conditions to surpass the confidence filter. Markers with higher abundance in late maturity (M3) in both cultivars are shown in Group 1. Those higher in early maturity (M1) in both cultivars are in Group 2. Groups 3 and 4 show markers higher in M3, only in ‘Chandler’ or ‘Tulare’, respectively. Groups 5 and 6 show markers higher in M1, only in ‘Chandler’ or ‘Tulare’, respectively. Complete lists of proteins with significant differential abundance are shown in Table S2.

A subset of 70 proteins in both with ‘Chandler’ and ‘Tulare’ nuts displayed consistently higher abundance in late maturity. The ones with the highest ratio fold-changes that can be considered good biomarkers for late maturity nuts include early nodulin-like protein-1 (Jr09_15040), glyoxysomal malate synthase (Jr02_01370), late embryogenesis abundant protein D-29 (Jr07_12690), BURP domain protein RD22 (Jr11_27400), dehydrin-1 (Jr08_18050), isocitrate lyase (Jr01_19240), endochitinase (Jr13_05720), and glucan endo-1,3-beta-glucosidase (Jr12_10420) (Figure 6). On the other extreme, there are 360 proteins with reduced abundance in late maturity in both cultivars. Prominent biomarkers include two 1-aminocyclopropane-1-carboxylate oxidases (Jr10_24060 and Jr04_21480), alanine-glyoxylate aminotransferase-2 (Jr04_00910), and squamosa promoter-binding protein-1 (Jr03_07500). In addition to these proteins of known function, 14 others yet uncharacterized also stand out as markers of early maturity, including Jr02_16400 and Jr04_13220 among the top.

Among the proteins with higher abundance in late maturity in ’Chandler’ pellicle tissue but not significantly different in ‘Tulare’, were four small heat shock proteins (Jr14_11390, Jr13_24070, Jr13_20730 and Jr07_37360), oleosin-5 (Jr01_04940), and a non-specific lipid-transfer protein AKCS9 (Jr01_07750). Those higher in ‘Tulare’ but not in ‘Chandler’ include chalcone synthase 1 and 2 (Jr12_09350 and Jr07_13470), non-specific lipid-transfer protein 1 (Jr03_26970), metal efflux detoxification protein (Jr15_06020), inositol oxygenase-4 (Jr01_27340), 12-oxophytodienoate reductase-2 (Jr10_01560), class V chitinase (Jr06_19660), and calcium-binding protein CML49 (Jr03_05020), among others. Proteins with higher abundance in early maturity in ‘Chandler’ but not significant in ‘Tulare’ include 2-oxoglutarate 3-dioxygenase (Jr11_07940), sugar transporter SWEET10 (Jr09_00400), asparagine synthetase (Jr14_08810), a third copy of 1-aminocyclopropane-1-carboxylate oxidase (Jr04_13540), and *ent*-kaurenoic acid oxidase (Jr14_02660). Complementing this comparison, proteins with higher abundance in early versus late maturity in ’Tulare’ but not significantly different in ‘Chandler’ include a class II heat shock protein (Jr06_10250), a protein of unknown function (Jr09_12260), biotin carboxyl carrier protein of acetyl-CoA carboxylase (Jr12_24840), glycerophosphodiester phosphodiesterase GDPD1 (Jr01_01230), and phospholipid glutathione peroxidase (Jr11_30350) (Figure 6).

The markers highlighted in Figure 6 are useful to differentiate the walnut fruit maturity stages but are only part of the whole proteome responsive to maturation. To gain a broader perspective on the molecular changes occurring among the maturity stages, gene ontology (GO) analysis was performed on the proteins with significant abundance differences between early and late maturity stages. Table 2 shows the most overrepresented terms in the proteome of mature nuts and Table 3 in immature nuts. Complete GO analysis results are listed in Table S3.

From the 70 proteins with increased abundance in both ‘Chandler’ and ‘Tulare’ nuts at late maturity, 61 were mapped to gene ontology terms. From the 360 with reduced abundance in late maturity in both cultivars, 315 were mapped to gene ontology terms. The statistical overrepresentation test is performed based on the difference between the observed and expected frequency of proteins annotated with a given GO term. The expected frequency considers all proteins with that annotation in the complete genome reference list (29,872 proteins with GO annotation), adjusted to the user input list size subjected to the analysis. This analysis reveals a clear distinction in pellicle metabolism between early and late maturity stages, with catabolic functions more evident in late maturity and biosynthetic functions and energy metabolism more evident in early maturity. The GO analysis also highlighted several amino acid biosynthesis pathways in the early maturity, such as tryptophan (P02783), leucine (P02749), isoleucine (P02748), and lysine (P02751). Energy metabolism, including TCA cycle (P00051), pyruvate metabolism (P02772), and glycolysis (P00024), also were overrepresented.

### 3.4. Comparison of Cultivars ‘Chandler’ and ‘Tulare’ Pellicle Proteome at Nut Harvest Stage

**Figure 7:**
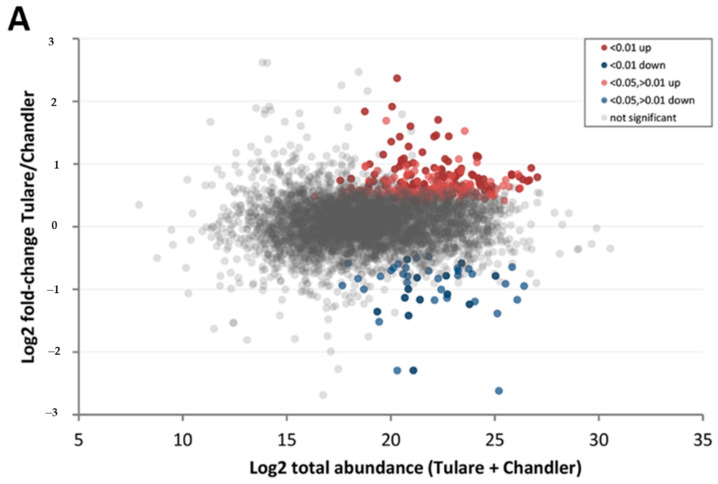

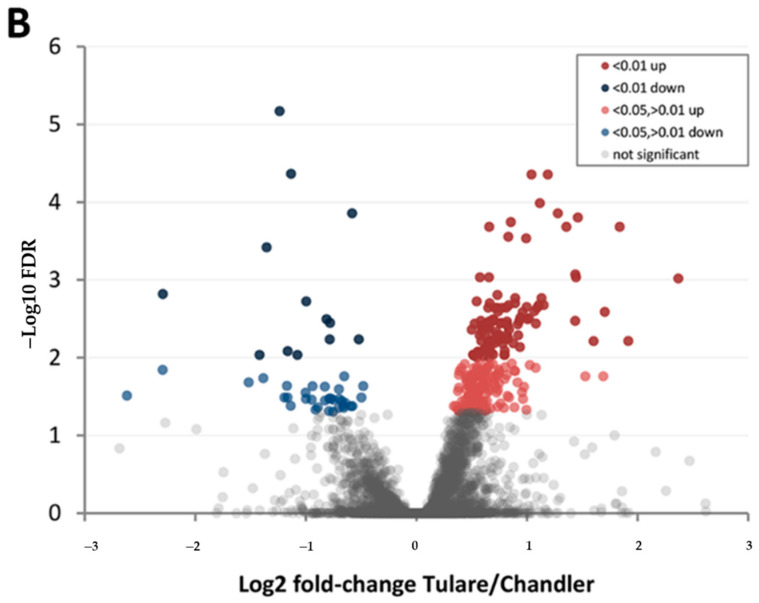
Visual representation of ‘Tulare’ and ‘Chandler’ proteomes compared at harvest stage. (**A**) Ratio vs. abundance plot of cultivar ‘Tulare’ compared to ‘Chandler’ at harvest (M2) stage, as listed in Table 1. Each dot represents the abundance of a protein, colored by the significance of the difference between ‘Tulare’ and ‘Chandler’. High confidence values (FDR < 0.01) are colored dark red (higher in ‘Tulare’) or dark blue (higher in ‘Chandler’), and medium confidence value (FDR < 0.05, >0.01) in lighter red and blue tones. Proteins without significant difference between cultivars are colored gray; (**B**) Volcano plot showing ‘Tulare’ vs. ‘Chandler’ fold-change ratios of proteome at harvest (M2) sorted by confidence levels, using the same color scheme.

In addition to samples at early and late maturity stages, our dataset also comprised samples at the intermediate stage of fruit maturation, M2. This stage is relevant as it represents most nuts at harvest. Given the higher frequency of nuts with extra light pellicle color (higher market value) in cultivar ‘Chandler’ compared to cultivar ‘Tulare’ (Figure 3), a direct comparison of pellicle proteomes at M2 maturity was performed. This analysis showed fewer proteins with significant differential abundance compared to the previous analysis between late and early maturities within each cultivar, whereas 93.5% of detected proteins showed similar abundance levels (Table 1). Still, 321 proteins showed differential abundance, 275 of them higher in ‘Tulare’ than in ‘Chandler’ (Figure 7). These include linamarin synthase 2 (Jr10_12470), MLP-like protein 28 (Jr13_23070), caffeic acid 3-O-methyltransferase (Jr16_05020), and a catalase isozyme (Jr11_18690) (Table S2). Interestingly, the protein with the highest fold-change abundance in cultivar ‘Tulare’ compared to ‘Chandler’ is of unknown function (Jr12_01470) and warrants further investigation.

**Figure 8:**
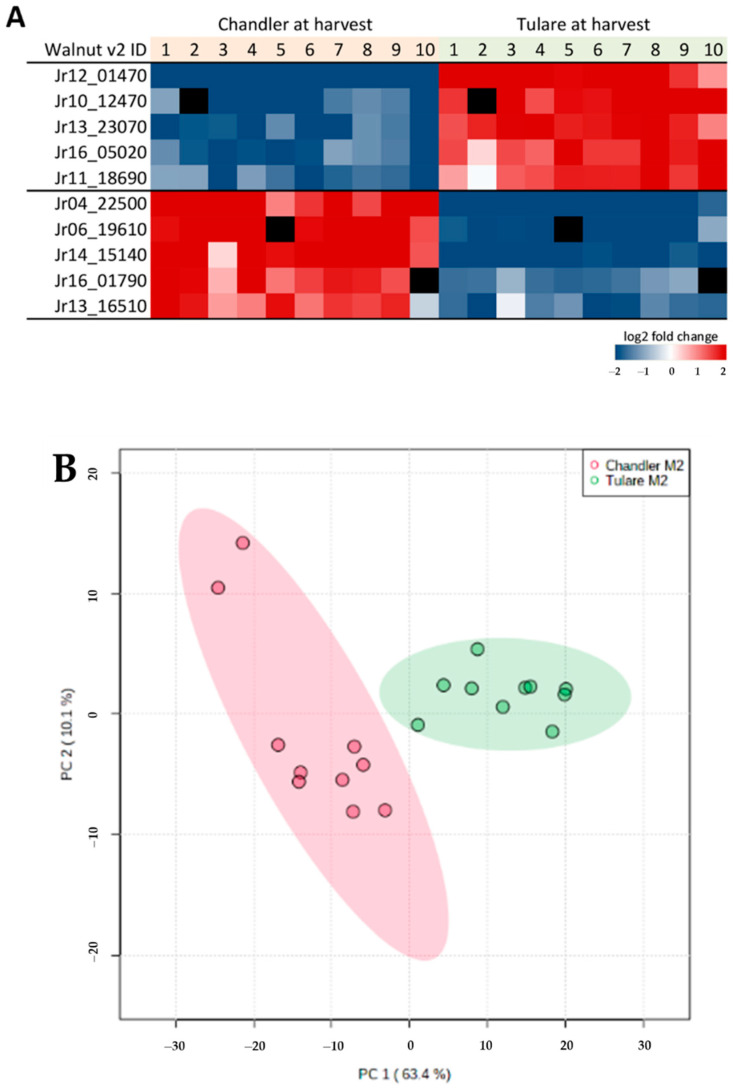
Selected proteins in pellicle tissue with differential abundance between ‘Chandler’ and ’Tulare’ cultivars at harvest stage. (**A**) Heatmap of protein biomarkers abundance ratios comparing the two cultivars at harvest stage (M2). The ten replicates for each of the two cultivars are shown, and individual ratios for each replicate were calculated using the average of the condition compared. Missing values for a particular replicate are shown in black. Protein markers with higher abundance ratios are shown in red and lower abundance ratios in blue. Complete lists of proteins with significant differential abundance are shown in Table S2. (**B**) PCA of ‘Chandler’ (red) and ‘Tulare’ (green) samples at harvest M2 maturity based on proteins with differential abundance. PC1 + PC2 explained 73.5% of the variance.

Among the 46 proteins with higher abundance in ‘Chandler’ relative to ‘Tulare’at harvest stage M2, thaumatin-like protein 1 (Jr04_22500), class V chitinase (Jr06_19610), beta-glucosidase BoGH3B (Jr14_15140), multicopper oxidase LPR2 (Jr16_01790), and glutathione S-transferase U17 (Jr13_16510) stand out (Figure 8A). The complete set is listed in Table S2. PCA analysis of M2 samples using the 321 proteins with differential abundance shows distinct clusters according to cultivar type (Figure 8B). Gene ontology analysis was also performed on the protein lists with differential abundance between cultivars ‘Tulare’ and ‘Chandler’, resulting in 250 and 45 mapped proteins with higher abundance in ‘Tulare’ and ‘Chandler’, respectively. The most overrepresented terms in cultivar ‘Tulare’ are shown in Table 4. The 46 proteins with higher abundance in ‘Chandler’ did not show overrepresented GO terms in any category, except PANTHER protein class, which highlighted “peroxidase” based on two hits from that list. The complete GO analysis results are shown in Table S3.

## 4. Discussion

Walnut pellicle physiology is critical for color development during kernel maturation. Previous studies have provided clues on critical proteins and metabolites playing roles in color development, such as sugar transferases and lipids [28,31,51]. Marrano and collaborators [28] used an SNP array to analyze QTLs involved with pellicle color, and proposed the participation of an UDP-glycosyltransferase located on chromosome 6. Although we did detect the expression of Jr06_12570, it did not figure among the top biomarkers in our dataset, either comparing the maturity stages or the genotypes. The metabolomics study by Rao [28] provided an insightful interpretation of carbon flow from carbohydrates and proteins towards lipids. These can then be used as an energy and carbon source for the germinating seed. Our data corroborate this notion, as ~80% of proteins with differential abundance between late and early maturity showed lower levels in M3. Moreover, we detected many enzymes involved in fatty acid and lipid metabolism. We imposed a minimum of two peptides mapped to any given protein to consider it as “detected”, which significantly decreases the number of identified proteins but increases the confidence of results. This conservative approach, allied with the ten replicate experiments, provides a robust dataset for biomarker discovery and overall understanding of the proteomes in each sample type. The protein abundances spanned six orders of magnitude, and among the ones with the highest abundance are seed storage proteins, vicilin-like antimicrobial proteins, lipid transfer proteins (LTP) and mannitol dehydrogenase; being a shared feature of both cultivars, among the three maturity stages analyzed (Table S1). Although not among the nut seeds, a soybean (*Glycine max* (L.) Merr. cv Jack) seed coat proteomic profiling detected many of these proteins on samples representing different maturity stages, as well as oxidoreductase enzymes, chaperones, and carbon flow towards phenylpropanoids [52]. This parallel shows that many processes are conserved on seed coat maturation even between plant species distantly related and forming very distinct seed types.

Of the 12 potential allergens detected by Marrano [30] in walnut pollen and catkins, eight were also detected here in the pellicle tissues, and all were among the most abundant proteins. We additionally detected four potential allergens in pellicle not detected in pollen or catkin: Jr01_32040 and Jr01_32060 (forming a cluster with Jr01_32050), Jr12_10760 and Jr12_10780 (forming a cluster with Jr12_10750). These can now be studied in further detail. In addition to playing roles in seed maturation, they also have antimicrobial activity [53,54,55,56,57,58] and might protect the kernel during maturation. LTPs are also classified as members of the pathogenesis-related protein 14 family (PR-14), important for plant defense [59]. The highly abundant sucrose-binding protein also plays a role in seed maturation [60]. When sorted by Gene ID, the protein abundance ranking reveals there are hotspots of expression and accumulation scattered throughout the genome, in all chromosomes. Many other highly abundant proteins are oxidoreductases, energy metabolism enzymes, and defense proteins such as chitinases.

Pellicle senescence is a process of intense metabolic activity promoting dehydration of the tissue and accumulation of proteins and smaller metabolites such as polyphenols. Many of these metabolic functions were mapped by the gene ontology analyses comparing the late (M3) vs. early (M1) maturity stages. The common themes observed in both cultivars delineate more general responses. A general shift from an intense biosynthetic metabolism to an overall arrest and activation of some catabolic functions shape the pellicle maturation process. All major classes of metabolites including carbohydrates, lipids, proteins and nucleic acids displayed activated biosynthetic functions at the M1 stage for both cultivars, but not in M3. The deep proteomic data generated captured this shift, already hinted at by merely observing the number of proteins with higher abundance in M1, 651, compared to those in M3, only 147. The detailed lists are available for further reference in Table S2, showing the abundance ratios between the two developmental stages. Glyoxysomal enzymes become more abundant in late maturity samples, with malate synthase and isocitrate lyase showing the greatest fold-change. This activation of the glyoxylate cycle bypass is indicative of regulation of carbon and nitrogen metabolism through fatty acids [61,62].

The biomarkers with the highest score (FDR and fold-change) present in each maturity stage pointed out in the Results section represent well this intense shift in metabolism as maturity progresses. For example, alanine-glyoxylate aminotransferase-2, a top marker for early maturity, supplies pyruvate to mitochondria assisting the high demand of this metabolite. Glutathione metabolism also is required to protect cells and enzymes from oxidative damage, feeding, for example, phospholipid hydroperoxide glutathione peroxidase, one of the top enzymes more abundant in early compared to late maturity pellicles. Other redox enzymes more prominent in early maturity include the 1-aminocyclopropane-1-carboxylate oxidases (ACC oxidases). A close inspection on all ACC oxidases revealed that a third copy (Jr07_23590) displays higher levels in cultivar ‘Tulare’ relative to ‘Chandler’. ACC oxidase is known to participate in ethylene signaling during seed development [63,64]. The higher abundance of enzymes of the methionine salvage/Yang cycle [65] corroborates this. Whether ’Tulare’ has a higher ethylene production and if this contributes to darker pellicles remains to be evaluated. One of two polyphenol oxidases (PPO) was detected on all samples, at similar but consistently medium-high levels (rank 1041 of 4937 proteins), also demonstrating the intense oxidative metabolism in pellicle tissue in both cultivars, but not a distinctive feature between the cultivars, at least not in these developmental stages. Interestingly, ‘Chandler’ accumulates many redox enzymes at higher levels than ‘Tulare’, possibly better coping with oxidative stress and preserving lighter pellicle color. A marked characteristic of ‘Chandler’ pellicles compared to ‘Tulare’ is also the higher abundance of several small heat shock proteins that possibly contribute to a less stressful desiccation process.

Besides the many other enzymes of intermediary, energy, and redox metabolism, some also caught our attention for providing critical clues on lipid metabolism in the pellicle tissue. These include the plastid-lipid-associated proteins, delta-(12)-acyl-lipid-desaturase, which is involved in the synthesis of a major fatty acid, linoleic acid [66], and the previously mentioned highly abundant lipid transfer proteins. Lipid metabolism towards late maturity provides hints on differences between ‘Tulare’ and ‘Chandler’, in which accumulation of oleosin-5 is only observed in the latter, being a useful marker for maturity stage in this cultivar and could be explicitly tested for pellicle color development in future studies. O-acyltransferase WSD1, involved in triacylglycerol, wax, and long-chain fatty acid biosynthesis, also follows this pattern, with higher abundance only in late maturity ‘Chandler’. Another difference between the cultivars is the higher carbon flow towards chorismate and flavonoids in ‘Tulare’, a major checkpoint controlling polyphenol pigment formation catalyzed by two chalcone synthases with higher abundance in ‘Tulare’. It also shows greater flow towards phenylpropanoids and indole alkaloids than ‘Chandler’, as can be attested by the higher abundance of caffeic acid 3-O-methyltransferase, phenylalanine ammonia-lyase, and vinorine synthase, for example. The extended metabolic activity seen in ‘Tulare’ also includes enzymes for sugar metabolism leading to D-glucose. Collectively these differences might provide enough substrate for pigment formation in later stages of kernel aging. Their degradation products can serve as reactants in the Maillard browning reaction [67] that occurs during processing and roasting.

Being able to map identified peptides on the whole proteome complement of *Juglans regia* enabled us to identify with high confidence many uncharacterized proteins featured among all analyses, including most abundant proteins, overrepresented according to maturity stage and genotype. These can now be investigated in more detail for unraveling their biochemical properties and contribution to pellicle maturation. Another great resource provided by the chromosome level genomic sequence [59] is the possibility to verify regulatory sequences present in promoter regions of selected protein-coding genes detected herein, thus capturing valuable information for machine learning of regulatory sequences. This resource might be used in future projects, for example, to guide gene-editing efforts to manipulate levels of desired proteins, without the need to recruit transgenes to achieve higher performance cultivars. This work contributes another step towards this knowledge base, in which the genomic sequence can be applied to precision breeding. More omics analyses will continue to unravel these processes both within and across cultivars.

## 5. Conclusions

Walnut kernel quality is determined by the pellicle tissue physiology leading to coloration. Using a combination of isobaric tagging of peptides, reversed-phase fractionation, conservative quality controls, expanded replicate design, and state of the art Orbitrap Lumos LC-MS/MS proteomics, we were able to investigate the pellicle maturation stages following PTB up to harvest in detail. Almost five thousand *Juglans regia* proteins we identified, spanning six orders of magnitude in individual abundance. In our proteomic analysis, the kernel color of ‘Chandler’ and ‘Tulare’ were very similar visually. However, their proteome revealed an intense shift from biosynthesis to catabolism along the three maturity stages. The elite cultivar ‘Chandler’ shows an extensive repertoire of antioxidants and chaperones that might better preserve the pellicle tissue integrity during desiccation. Alternatively, cultivar ‘Tulare’ sustains a prolonged metabolic activity and accumulation of proteins. Many proteins involved in secondary metabolism, including pigment formation, showed higher abundance in ‘Tulare’, as well as enzymes involved in ethylene synthesis and precursors of Maillard browning reaction that can develop later during commercial drying. A follow up to this work can include investigations in later developmental stages, in which most of the pellicle coloring develops. Molecular studies on pellicle coloring may provide candidate genes for precision breeding efforts that will add value to these cultivars.

## Figures and Tables

**Table 1:** Proteins with differential abundance in the two cultivars detected in pellicle tissue during maturation process.

Protein Category	‘Chandler’ M3/M1 ^1^	‘Tulare’ M3/M1	‘Tulare’/’Chandler’ M2
FDR < 0.01 up	66	76	103
FDR < 0.05, >0.01 up	114	71	172
FDR < 0.01 down	241	388	13
FDR < 0.05, >0.01 down	374	263	33
Difference not significant	4142	4139	4616

^1^ M1, M2, and M3 refer to early, intermediate, and late maturity stages, respectively. False Discovery Rate (FDR) values obtained from the Benjamini-Hochberg correction for multiple comparisons.

**Table 2:** Gene ontology terms overrepresented ^1^ in late vs. early maturity walnut pellicle proteome.

PANTHER GO-Slim Biological Process	Observed	Expected	Fold Change	*p*-Value	FDR
secondary metabolic process (GO:0019748)	5	0.08	66.18	2.39 × 10^−8^	3.57 × 10^−5^
proteolysis (GO:0006508)	7	1.17	5.96	1.78 × 10^−4^	3.33 × 10^−2^
cellular catabolic process (GO:0044248)	8	1.59	5.04	1.86 × 10^−4^	3.09 × 10^−2^
**PANTHER GO-Slim Molecular Function**					
carboxypeptidase activity (GO:0004180)	6	0.12	50.66	3.80 × 10^−9^	1.69 × 10^−6^
hydrolase activity (GO:0016825)	6	0.21	28.25	1.01 × 10^−7^	1.50 × 10^−5^
acyl transferase activity (GO:0016746)	6	0.45	13.42	6.64 × 10^−6^	4.92 × 10^−4^
**PANTHER GO-Slim Cellular Component**					
anchored on plasma membrane (GO:0046658)	4	0.22	17.97	8.51 × 10^−5^	3.49 × 10^−2^
**PANTHER Protein Class**					
serine protease (PC00203)	6	0.54	11.13	1.87 × 10^−5^	3.19 × 10^−3^

^1^ PANTHER Statistical overrepresentation test of gene ontology term. No statistically significant results were detected for category PANTHER Pathways.

**Table 3:** Gene ontology terms overrepresented in early vs. late maturity walnut pellicle proteome.

PANTHER GO-Slim Biological Process	Observed	Expected	Fold Change	*p*-Value	FDR
branched-chain amino acid biosynthesis (GO:0009082)	5	0.13	39.51	6.69 × 10^−7^	2.33 × 10^−5^
glycolytic process (GO:0006096)	8	0.41	19.45	2.84 × 10^−8^	1.09 × 10^−6^
aromatic amino acid family biosynthesis (GO:0009073)	4	0.26	15.17	2.25 × 10^−4^	4.26 × 10^−3^
transmembrane import into organelle (GO:0044743)	5	0.35	14.37	4.54 × 10^−5^	1.01 × 10^−3^
tricarboxylic acid cycle (GO:0006099)	3	0.23	12.93	2.18 × 10^−3^	3.14 × 10^−2^
chaperone-mediated protein folding (GO:0061077)	6	0.52	11.61	2.32 × 10^−5^	5.88 × 10^−4^
purine ribonucleotide biosynthetic process (GO:0009152)	5	0.71	7.08	9.47 × 10^−4^	1.56 × 10^−2^
cofactor biosynthetic process (GO:0051188)	5	0.93	5.39	2.95 × 10^−3^	3.83 × 10^−2^
translational elongation (GO:0006414)	10	3.35	2.98	2.51 × 10^−3^	3.44 × 10^−2^
**PANTHER GO-Slim Molecular Function**					
mRNA 3′-UTR binding (GO:0003730)	2	0.08	23.71	4.62 × 10^−3^	4.03 × 10^−2^
heat shock protein binding (GO:0031072)	5	0.27	18.24	1.63 × 10^−5^	2.79 × 10^−4^
ATP binding (GO:0005524)	5	0.41	12.16	9.32 × 10^−5^	1.38 × 10^−3^
endopeptidase activity (GO:0004175)	11	1.96	5.61	7.86 × 10^−6^	1.52 × 10^−4^
ligase activity (GO:0016874)	6	1.22	4.91	1.81 × 10^−3^	1.87 × 10^−2^
**PANTHER GO-Slim Cellular Component**					
proteasome core complex (GO:0019773)	5	0.08	59.27	1.44 × 10^−7^	3.69 × 10^−6^
respiratory chain complex I (GO:0045271)	3	0.32	9.48	4.87 × 10^−3^	4.34 × 10^−2^
mitochondrial matrix (GO:0005759)	4	0.5	8.07	1.98 × 10^−3^	2.19 × 10^−2^
plastid part (GO:0044435)	7	1.78	3.93	2.59 × 10^−3^	2.59 × 10^−2^
cytosolic ribosome (GO:0022626)	9	2.43	3.71	9.74 × 10^−4^	1.29 × 10^−2^
**PANTHER Protein Class**					
chaperonin (PC00073)	8	0.2	39.93	2.40 × 10^−10^	8.22 × 10^−9^
acetyltransferase (PC00038)	4	0.3	13.55	3.32 × 10^−4^	4.06 × 10^−3^
epimerase/racemase (PC00096)	3	0.26	11.38	3.04 × 10^−3^	3.25 × 10^−2^
isomerase (PC00135)	7	0.62	11.25	5.79 × 10^−6^	9.91 × 10^−5^
chaperone (PC00072)	14	1.31	10.71	2.25 × 10^−10^	9.60 × 10^−9^
dehydrogenase (PC00092)	17	2.35	7.23	7.56 × 10^−10^	2.15 × 10^−8^
transaminase (PC00216)	4	0.56	7.16	2.97 × 10^−3^	3.39 × 10^−2^
translation factor (PC00223)	8	1.21	6.6	4.69 × 10^−5^	6.68 × 10^−4^
lyase (PC00144)	12	2.34	5.13	7.27 × 10^−6^	1.13 × 10^−4^

**Table 4:** Gene ontology terms over-represented in the pellicle proteome of cultivar ‘Tulare’ vs. ‘Chandler’.

PANTHER GO-Slim Biological Process	Observed	Expected	Fold Change	*p*-Value	FDR
tricarboxylic acid cycle (GO:0006099)	4	0.18	21.73	6.00 × 10^−5^	1.72 × 10^−3^
reactive oxygen species metabolic process (GO:0072593)	5	0.25	19.91	1.00 × 10^−5^	4.28 × 10^−4^
glycolytic process (GO:0006096)	5	0.33	15.32	3.15 × 10^−5^	1.07 × 10^−3^
protein targeting to mitochondrion (GO:0006626)	5	0.37	13.58	5.36 × 10^−5^	1.60 × 10^−3^
**PANTHER GO-Slim Molecular Function**					
ATP binding (GO:0005524)	4	0.33	12.26	4.43 × 10^−4^	7.59 × 10^−3^
unfolded protein binding (GO:0051082)	10	0.98	10.21	1.16 × 10^−7^	1.03 × 10^−5^
endopeptidase activity (GO:0004175)	11	1.56	7.07	8.70 × 10^−7^	4.30 × 10^−5^
small molecule binding (GO:0036094)	13	1.92	6.75	1.43 × 10^−7^	1.06 × 10^−5^
oxidoreductase activity (GO:0016491)	19	7.64	2.49	3.17 × 10^−4^	5.89 × 10^−3^
**PANTHER GO-Slim Cellular Component**					
proteasome core complex (GO:0019773)	6	0.07	89.62	8.74 × 10^−10^	7.17 × 10^−8^
**PANTHER Protein Class**					
peroxidase (PC00180)	3	0.11	27.57	2.92 × 10^−4^	6.24 × 10^−3^
isomerase (PC00135)	5	0.49	10.13	1.94 × 10^−4^	4.73 × 10^−3^
lyase (PC00144)	13	1.86	7	9.71 × 10^−8^	3.32 × 10^−6^
dehydrogenase (PC00092)	11	1.87	5.89	4.62 × 10^−6^	1.32 × 10^−4^
oxidoreductase (PC00176)	29	8.85	3.28	3.57 × 10^−8^	1.52 × 10^−6^
**PANTHER Pathways**					
Glycolysis (P00024)	4	0.19	20.78	7.00 × 10^−5^	3.75 × 10^−3^

## Supplementary Materials

The following are available online at https://www.mdpi.com/2075-1729/10/12/314/s1, Table S1: Proteomics dataset showing intensity abundance for individual proteins in the 60 samples. Table S2: Complete lists of proteins with significant differential abundance. Table S3: Gene ontology analysis of proteins with differential abundance.
